# Myocardial Injury During the Immediate Postoperative Period After Endovascular Aortic Repair: A Prospective Observational Study

**DOI:** 10.3390/bioengineering13020185

**Published:** 2026-02-05

**Authors:** Manolis Abatzis-Papadopoulos, Konstantinos Tigkiropoulos, Christina Antza, Christina Alexou, Anthi-Maria Lazaridi, Katerina Sidiropoulou, Kyriakos Stavridis, Dimitrios Karamanos, Vasilios Kotsis, Ioannis Lazaridis, Nikolaos Saratzis

**Affiliations:** 1 Surgical Department, General Hospital of Katerini, 60100 Katerini, Greece; 2 Vascular Unit, 1st University Surgical Department, Papageorgiou General Hospital, School of Medicine, Aristotle University of Thessaloniki, 56403 Thessaloniki, Greece; kostastig@yahoo.com (K.T.); kasidi94@hotmail.com (K.S.); kstavridis17@yahoo.gr (K.S.); dkaramanos@gmail.com (D.K.); drlazaridis@yahoo.gr (I.L.); nicos_saratzis@yahoo.com (N.S.); 3 3rd University Department of Internal Medicine, Papageorgiou General Hospital, School of Medicine, Aristotle University of Thessaloniki, 56403 Thessaloniki, Greece; kris-antza@hotmail.com (C.A.); vkotsis@auth.gr (V.K.); 4 Cardiothoracic Surgery Department, Papanikolaou General Hospital, 57010 Thessaloniki, Greece; christinaalexou@gmail.com; 5 Primary Care Center of Ptolemaida, General Hospital of Ptolemaida “Mpodosakeio”, 50200 Ptolemaida, Greece; anthilazaridi@gmail.com

**Keywords:** abdominal aortic aneurysm, aortic stiffness, endovascular aortic repair, aortic endograft, myocardial injury, high sensitivity troponin

## Abstract

Background/Objectives: Several studies in the literature support that endovascular aortic repair (EVAR) could exert a harmful effect on heart function, causing myocardial injury. The aim of this study is to investigate the correlation of high-sensitivity cardiac troponin I (hs-cTnI) levels during the immediate postoperative period after EVAR with various factors. Methods: A total of 104 patients were enrolled from February to December 2024 prospectively and consecutively. Patient demographics, cardiovascular comorbidities, laboratory tests, including hemoglobin and hs-cTnI levels, EVAR procedure duration and type of anesthesia were recorded. A generalized linear mixed model with a Gamma distribution and log link was fitted to analyze postoperative hs-cTnI concentrations across three time points (6 h, 24 h, and 48 h). Results: The mean age of patients was 71.6 ± 7.3 years, the mean transverse AAA diameter was 5.7 ± 1.1 cm and the mean preoperative hemoglobin was 14.2 ± 1.64 g/dL. In total, 72 patients received general anesthesia and 32 patients regional anesthesia. A total of 18 patients presented myocardial injury. Patients under general anesthesia had significantly higher mean hs-cTnI than those under regional anesthesia (*p* < 0.01). Older age, longer operations, and higher baseline hs-cTnI all predict higher follow-up hs-cTnI. Meanwhile higher preoperative hemoglobin predicts lower hs-cTnI. Conclusions: General anesthesia compared to regional anesthesia, older age, longer surgery, and higher baseline hs-cTnI are associated with higher postoperative hs-cTnI levels, while higher preoperative hemoglobin predicts lower hs-cTnI levels. Understanding the factors that are related to myocardial injury during EVAR could contribute to the improvement in procedures in order to minimize the harmful effect of EVAR on heart function.

## 1. Introduction

It has been more than 35 years since the first successful endovascular aortic repair (EVAR) for abdominal aortic aneurysm (AAA) [1]. In the modern era of AAA management, EVARs have outnumbered open surgical repair (OSR). The main reason for this is the early benefits of EVARs on patient morbidity and mortality compared to OSR. These findings are supported by the European Society of Vascular Surgery (ESVS) guidelines regarding the management of AAAs [2]. However, EVAR has been related to major adverse cardiovascular events (MACEs) by several studies in the literature [3]. Various studies support the proposal that EVAR could have a harmful effect on myocardial function, even causing myocardial injury [4], while others try to study the predictors of mortality after EVAR [5].

High-sensitivity troponin (hs-cTn) is a blood marker used in the detection of myocardial injury or stress. The available assays are so sensitive that they can detect small amounts of troponin in patient plasma, which are usually detected in subclinical myocardial injury or stress, while hs-cTn levels are markedly increased in myocardial infarction [6]. Furthermore, increased levels of hs-cTn are strong predictors of future events, such as major adverse cardiovascular events [7,8].

Several studies in the literature have demonstrated an increase in hs-cTn in EVAR patients, implying that EVAR could cause subclinical myocardial injury, which is related to adverse outcomes in the postoperative period [5,9]. The aim of this study is to investigate the fluctuations in hs-cTn levels in EVAR patient plasma during the immediate postoperative period and its correlation with various causing factors, such as the duration of the operation, the type of anesthesia and patient characteristics (e.g., age and preoperative hemoglobin levels).

## 2. Materials and Methods

### 2.1. Study Design

The study had a prospective observational design. All eligible patients, based on the inclusion criteria, were enrolled following a prospective and consecutive method from February 2024 to December 2024. Informed written consent was obtained from all patients enrolled in our study. Detailed medical histories of patients were obtained and their demographics, laboratory tests (including hemoglobin and hs-cTn levels), and operation details including EVAR duration and type of anesthesia were recorded. Cardiovascular comorbidities before EVAR and cardiovascular adverse events and complications during EVAR and during the immediate postoperative period after EVAR were recorded in detail.

### 2.2. Sample Size

According to the effect size suggested by Sousa et al. [4], we used a fixed model to conduct our power analysis regarding the sample size of the study required for the analysis of our main findings. Statistical significance was considered for a *p*-value < 0.05 and the power level was set at 80%. In order to study the effect of the four factors of our study, we found that 104 patients were required without any dropouts. In the event of a dropout, an extra patient was enrolled in the study in order to achieve the sample size required for our analysis.

### 2.3. Inclusion/Exclusion Criteria

The inclusion criteria were as follows: patients who were adults of either male or female gender, suffered from infrarenal AAAs with the threshold for EVAR management a maximum transverse diameter of at least 55 mm in men and 50 mm in women and/or a common iliac artery aneurysm (CIA) with a maximum transverse diameter of at least 40 mm, managed electively with standard EVAR using aortobiiliac devices by implementing the ESVS guidelines regarding the management of AAAs and CIAs; hs-cTn was measured preoperatively and for at least the first 24 h postoperatively after EVAR.

Meanwhile, patients were excluded from our study if they suffered from complex aneurysms and were managed with fenestrated and branched devices or with parallel graft techniques, patients who were managed using devices other than the aortobiiliac endografts used in standard EVAR. We excluded patients with ruptured or inflammatory AAAs and patients with connective tissue diseases, as they could present deteriorated heart function even before EVAR due to the primary disease. We also excluded patients with end-stage renal disease (ESRN), as hs-cTn could be affected by ESRN and patients who have already been subjected to any intervention on the aorta. Moreover, patients without adequate recording of hs-cTn pre- and post-EVAR were excluded from our study.

### 2.4. Definition of Variables

The endpoints of our study were the correlations of any changes in hs-cTn levels before and after EVAR and various causal factors, such as the duration of the operation, the type of anesthesia and patient characteristics (including their age and preoperative hemoglobin levels).

The type of hs-cTn measured was type I (hs-cTnI). hs-cTnI was measured using the cTnI assay from Abbott Diagnostics (ARCHITECT STAT high-sensitivity Troponin, Abbott Diagnostics, Lake Forest, IL, USA). The manufacturer set the limit of detection at 3.4 pg/mL (range, 0–50,000 pg/mL) and the 99th percentile concentrations of healthy men and women are set at 34.2 and 15.6 pg/mL, respectively [10]. Myocardial injury was defined as any measurement of hs-cTnI exceeding these levels. According to European Society of Cardiology any hs-cTnI value of ≥64 pg/mL was considered high and set high suspicion of myocardial infarction diagnosis [11]. This cutoff level has been set by large studies regarding the assay used in our institution and its positive predictive value is about 75% [12,13]. hs-cTnI levels were measured in patient plasma preoperatively and at 6 and 24 h postoperatively after EVAR. Patients not discharged on the first postoperative day were investigated for their hs-cTnI levels at 48 h too. In cases of increased hs-cTnI, a cardiologist assessment was ordered. Even in cases of high hs-cTnI levels, the diagnosis of myocardial infarction and further management of patients was conducted by cardiologists, as a high value of hs-cTnI is not absolutely diagnostic of myocardial infarction and presents a 75% positive predictive value. MACEs were defined either as myocardial infarction with/without the need for revascularization or cardiovascular death postoperatively.

AAA and CIA diameters were measured by studying preoperative CTAs of patients with a 3mensio vascular workstation (Pie Medical Imaging BV, Maastricht, The Netherlands). Maximum transverse diameters were calculated by the outer-to-outer method by a vascular surgeon and a radiologist and inter-observer differences were solved by a measurement by a third physician. The duration of the operation was measured in minutes and was defined as the time between the first cut and the last suture during the EVAR procedure. The patients were subjected to EVAR under either general or regional anesthesia. All patients in the regional anesthesia group received spinal anesthesia. The type of anesthesia used during the EVAR procedure was studied as a possible causative factor for hs-cTnI fluctuations postoperatively. The type of anesthesia administered to each patient was decided upon by the anesthesiologists. Hemoglobin levels were measured in g/dL. Although the levels of hemoglobin were recorded pre and postoperatively, only preoperative values were used for the purposes of our study in order to investigate any correlation between preoperative hemoglobin and hs-cTnI levels fluctuations in patient plasma.

### 2.5. Surgical Procedure

All patients included in our study were subjected to elective EVAR. The procedures were conducted in an operative suite and a C-arm device was used for fluoroscopy during EVAR. Aortobiiliac endografts were used in our study and extension to the external iliac artery was performed in the absence of an adequate iliac artery landing zone and upon the surgeons’ decision by preserving flow to a single internal iliac artery. The preservation of internal iliac arteries was achieved by an iliac branch endoprostheses deployment or, in case of decision on preservation of one of the two iliac arteries, a plug was released in the embolised internal iliac artery with combined ipsilateral extension to external iliac artery. The contrast media administered for EVAR procedure was Ultravist^®^ Iopromide 300 mg/mL Injection 100 mL (Bayer Pharma AG, Berlin, Germany). The endografts used in our study were manufactured using a metallic skeleton compounded with nitinol or other alloy metal stents and a fabric material which was either Dacron polyester or expanded polytetrafluoroethylene. Patients were usually discharged on the first or second postoperative day after a technically successful EVAR and an uneventful postoperative course.

### 2.6. Data Collection

All data regarding the studied factors were collected and recorded in spreadsheets so that further analysis could be conducted. These data regarded patient demographics, full medical history, comorbidities, (especially heart diseases), blood investigations (including hemoglobin and hs-cTnI levels), medications, daily habits (including smoking and alcohol consumption), the duration of the operation and the type of anesthesia.

### 2.7. Statistical Synthesis and Analysis

A generalized linear mixed model with a Gamma distribution and log link was fitted to postoperatively analyze hs-cTnI concentrations across three time points (6 h, 24 h, and 48 h). Fixed effects included anesthesia type, time, surgery length, age, preoperative hemoglobin, and baseline hs-cTnI, as well as anesthesia by time interactions. A random intercept accounted for within-subject correlation. All covariates were statistically significant (*p* < 0.001) and the analysis was conducted using Jamovi v.2.6.13.0.

## 3. Results

### 3.1. Patient Demographics and Comorbidities

In total, 146 patients with aneurismal disease were subjected to endovascular repair. 42 patients were rejected according to the exclusion criteria. A total of 104 patients were enrolled in our study in a consecutive and prospective way. All patients met the inclusion criteria, suffered from infrarenal AAA and managed it electively with EVAR. The mean age of patients was 71.6 ± 7.3 years, 93.3% were males and 6.7% were females. The mean body mass index (BMI) of patients was 26.6 ± 3.3 kg/m^2^, active smokers were 51.9% of the patients, while 48.1% of the patients had quit smoking. Table 1 presents the demographics and daily habits of the patients enrolled in our study.

**Table 1:** Patient demographics and daily habits.

Demographic	Finding
Number of patients, n	104
Males:Females, n:n (%:%)	97 (93.3%):7 (6.7%)
Age, years (±SD)	71.6 (±7.3)
BMI, kg/m^2^ (±SD)	26.6 (±3.3)
Smokers: Active, n (%)	54 (51.9)
Previous, n (%)	50 (48.1)
Alcohol: Yes, n (%)	53 (51)
No, n (%)	51 (49)

n: number; SD: standard deviation; BMI: body mass index.

Table 2 presents comorbidities and medications of the patients included in our study. Regarding the heart comorbidities, 36 patients in our study suffered from coronary artery disease (CAD), 20 had suffered from acute coronary syndrome (ACS), 23 had been subjected to percutaneous coronary intervention (PCI), 7 had been subjected to coronary artery bypass grafting (CABG), 2 suffered from heart failure (HF) and 10 patients suffered from atrial fibrillation (AF). Regarding other comorbidities, 71.2% of the patients suffered from hypertension, 75% from dyslipidemias, 18.3% from diabetes mellitus, 15.4% from chronic obstructive pulmonary disease (COPD), 67.3% from chronic kidney disease (CKD), 14.4% from stroke. In total, 63 patients were receiving antiplatelets agents, 12 patients were receiving anticoagulants and 68 patients were receiving statins.

### 3.2. Main Findings

The mean transverse AAA diameter of the patients was 5.7 ± 1.1 cm. The endografts implanted were Ankura (Lifetech Scientific, Shenzen, China) in 71 patients (68.2%), Endurant II (Medtronic Inc., Minneapolis, MN, USA) in 10 patients (9.7%), Gore Excluder (W. L. Gore & Associates, Inc., Flagstaff, AZ, USA) in 9 patients (8.7%), Alto (Endologix Inc., Irvine, CA, USA) in 6 patients (5.8%), Jotec (Jotec GmbH, Hechingen, Germany) in 5 patients (4.8%) and Zenith Alpha (Zenith Alpha AAA, Cook, Inc., Bloomington, IN, USA) in 3 patients (2.9%). An extension to the external iliac artery was deployed in 10 patients (9.6%). A total of 72 patients received general anesthesia and 32 patients received regional anesthesia. Preoperative hemoglobin was 14.2 ± 1.6 g/dL. The mean operation time was 92.6 ± 25.4 min, the mean radiation time was 7.4 ± 4.7 min and the mean contrast quantity used was 107.1 ± 63.3 ml. Table 3 presents the properties of the endografts implanted and the operation details of all patients included in our study.

In total, 18 patients (17.3%) presented myocardial injury during the postoperative period. 15 patients received general anesthesia, while the remaining 3 received regional anesthesia. In two male patients (1.9%), one received general anesthesia and one regional anesthesia, presented symptoms and ECG findings. These patients were transferred to the cardiac care unit, managed further by cardiologists conservatively and were discharged after a few days. No deaths were recorded during the hospitalization of all included patients in our study. Table 4 presents the respective findings.

**Figure 1:**
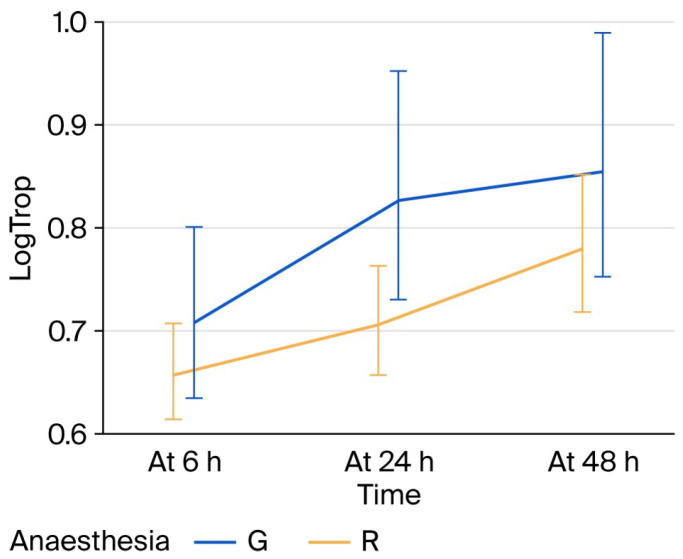
hs-cTnI fluctuations at 6, 24 and 48 h postoperatively in the two anesthesia groups. LogTrop: log link of high-sensitivity troponin I; h: hours; G: general, R: regional.

After adjusting for baseline hs-cTnI, preoperative hemoglobin, operation duration, and age, mean hs-cTnI levels were 35% higher at 24 h and 65% higher at 48 h compared with 6 h in patients under regional anesthesia. Patients receiving general anesthesia had 46% higher mean hs-cTnI values at 6 h, and the excess increase was 34% higher at 24 h and 15% higher at 48 h relative to regional anesthesia. Post hoc pairwise comparisons confirmed that hs-cTnI increased significantly over time in both anesthesia groups. In regional anesthesia, mean hs-cTnI increased 136% from 6 h to 24 h and 92% from 6 h to 48 h. In general anesthesia, mean hs-cTnI increased 89% from 6 h to 24 h and 76% from 6 h to 48 h. At each time point, patients under general anesthesia had significantly higher mean hs-cTnI than those under regional anesthesia: 121% higher at 6 h, 66% higher at 24 h, and 56% higher at 48 h (all Bonferroni- and Tukey-adjusted *p* < 0.001). These findings confirm the significant Anesthesia × Time interaction observed in the main model. Table 5 and Figure 1 present the findings regarding the effect of the type of anesthesia on hs-cTnI levels arithmetically and graphically by bar charts, respectively.

**Figure 2:**
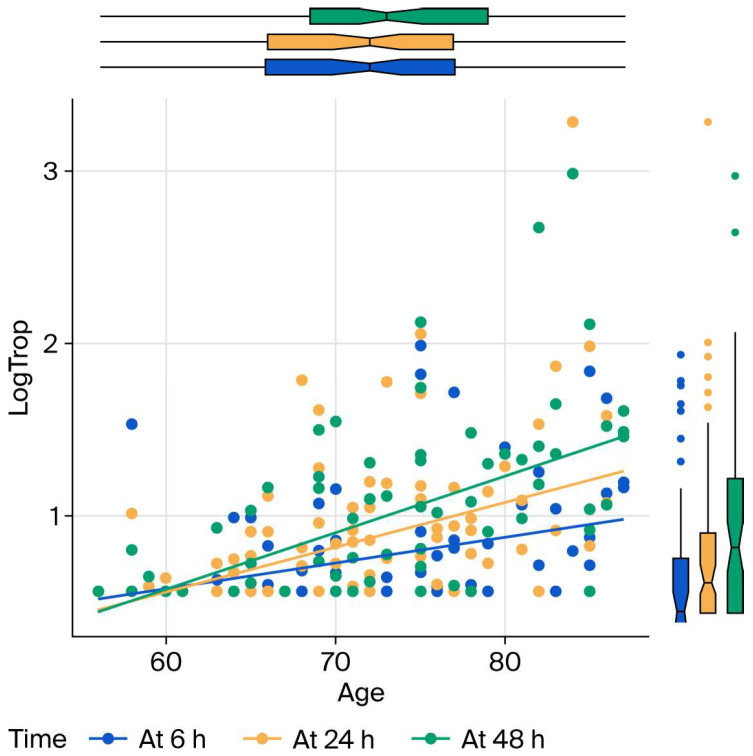
Effect of age on hs-cTnI levels at 6, 24 and 48 h postoperatively. LogTrop: log link of high-sensitivity troponin I; h: hours.

**Figure 3:**
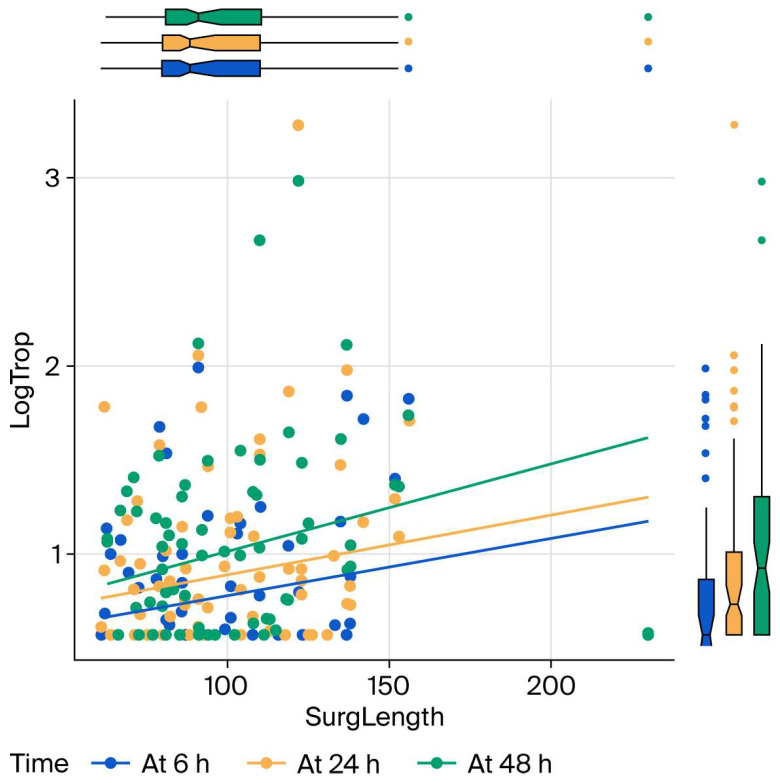
Effect of surgery duration on hs-cTnI levels at 6, 24 and 48 h postoperatively. LogTrop: log link of high-sensitivity troponin I; h: hours; SurgLength: duration of the surgery.

**Figure 4:**
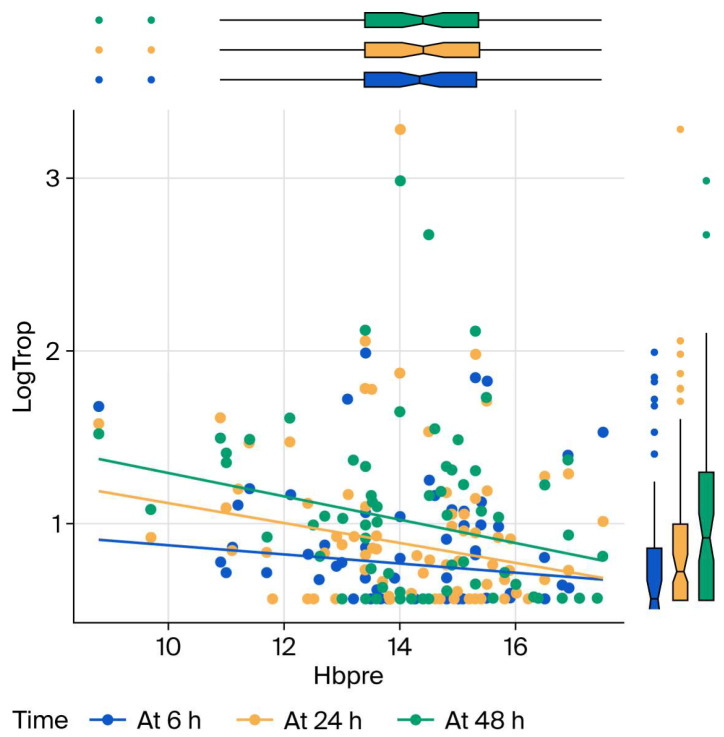
Effect of preoperative hemoglobin on hs-cTnI levels at 6, 24 and 48 h postoperatively. LogTrop: log link of high-sensitivity troponin I; h: hours; Hbpre: preoperative hemoglobin.

Older age, longer operations, and higher baseline hs-cTnI all predict higher follow-up hs-cTnI; higher preoperative hemoglobin predicts lower hs-cTnI. These results are presented in Figure 2, Figure 3 and Figure 4 as charts. Specifically, each additional 10 min in surgical duration was associated with a 5% increase in mean hs-cTnI (95% CI: 3–7%). Each additional year of age was associated with a 4.3% increase in mean hs-cTnI (95% CI: 4.1–4.4%). Each unit increase in preoperative hemoglobin was associated with a 0.6% decrease in mean troponin (95% CI: 0.5–0.8%). Each unit increase in baseline hs-cTnI was associated with a 1.5% increase in postoperative mean hs-cTnI (95% CI: 1.3–1.7%).

## 4. Discussion

In the modern era of AAA management, EVAR remains the preferred choice for AAA treatment over OSR due to its lower morbidity and mortality rates during the immediate postoperative period [2]. However, EVAR has been related to MACEs by several studies in the literature [3].

At the same time, more and more studies are being published describing the negative effect of EVAR procedure on various organ targets, and especially the heart [4,9]. One reliable marker of myocardial injury is hs-cTnI levels in patient plasma. The available assays can detect small amounts of troponin, which can be related to subclinical myocardial injury and stress [6]. Moreover, these increases in hs-cTnI have been related to future adverse cardiovascular events, even though these increases remain subclinical at the time they are measured [7].

In our study we investigated the effects of various factors on postoperative hs-cTnI fluctuations during the first 48 h after EVAR. We found that the type of anesthesia, the duration of the operation, the age, and preoperative hemoglobin levels of patients can exert an influence on postoperative hs-cTnI levels in patient plasma after EVAR.

In our study general anesthesia is related to greater increases in hs-cTnI than regional anesthesia postoperatively after EVAR. This difference between the types of anesthesia is observed at all postoperative time points. It is generally accepted that regional anesthesia is related to lower risk of myocardial injury, as surgery can elevate sympathetic activity and oxidative stress, while regional anesthesia can protect the heart from these injuries [14,15]. A large pool of patients subjected to operations with regional anesthesia comprises patients with hip fractures. In their meta-analysis of randomized studies, Lin et al., found that spinal anesthesia was associated with a lower risk of intraoperative hypotension and lower doses of ephedrine in older patients undergoing hip fracture surgery. Hypotension is considered a main causative factor for myocardial injury during an operation [16]. Similarly, Chen et al. in their meta-analysis, including observational studies apart from randomized ones, found that regional anesthesia is associated with improved perioperative outcomes in patients undergoing hip fracture surgery [17]. Even spinal anesthesia has been related to myocardial injury in some studies, as spinal sympathetic block and consequent vasodilation could cause sudden hypotension. However, there are fewer of these studies and they are mainly limited to descriptions of case reports [18]. In their study of EVAR patients, Sousa et al. [4] did not find any difference in myocardial injury between patients who received general and regional anesthesia, in contrast to our findings. However, this study was retrospective and patients were included if they had at least one troponin measurement in the first 48 postoperative hours after EVAR. Under this circumstance, some cases of subclinical myocardial injury might be lost, resulting in these different findings compared to our findings [4].

Additionally, in our study we found that the fluctuations in hs-cTnI were higher during the first postoperative day compared to the second one. This finding could be attributed to the higher stress on the heart during the first hours immediately after the operation. While most patients with increased hs-cTnI during the first postoperative day continued to show rising hs-cTnI levels during the second postoperative day, this rise was attenuated, likely showing a decrease in the heart stress. Marketou et al. found early left ventricular global longitudinal strain deterioration after AAA repair, affecting the systolic function of the heart negatively even during the first postoperative days [19].

Furthermore, we found that older age and longer operations cause higher hs-cTnI levels postoperatively and are related to myocardial injury, while higher preoperative hemoglobin is related to lower postoperative increases in hs-cTnI levels after EVAR. The findings of several studies in the literature agree with our results regarding these causative factors for myocardial injury. Duceppe et al. and Moussa et al. demonstrated that age and duration of operation are independent factors for myocardial injury during EVAR, while increased hemoglobin levels play a protective role for heart stress [20,21]. Sousa et al. stated that a cutoff of 12 g/dL in hemoglobin levels defines the threshold for myocardial injury caused during EVAR [4]. Additionally, in the present study we found that increased hs-cTnI baseline levels are related to an increased risk of myocardial injury postoperatively after EVAR. This is generally attributed to the comorbidities these patients suffered from, aside from the operation. These comorbidities mainly concerned heart diseases and played a major role in hs-cTnI level increases after the operative stress.

In general, we found that 17.3% of EVAR patients presented myocardial injury during the immediate postoperative period. The respective findings of Sousa et al., Duceppe et al. and Moussa et al. were 16.2%, 29% and 42.9% [4,20,21]. In their meta-analysis, Francisco-Azevedo et al. found that myocardial injury after EVAR ranged from 0.4% to 18.7%. The finding for OSR ranged from 1.8% to 46.8%, which is increased compared to EVAR at least in the immediate postoperative period [22]. By taking into account the findings of our study along with the published literature, patients who are older and present more serious comorbidities should be subjected to EVAR under regional anesthesia, as AAA patients managed with elective EVAR under regional anesthesia presented lower risk for myocardial injury. Before elective EVAR, special care should be taken for adequate hemoglobin levels of AAA patients, as low haemoglobin levels could be investigated and treated properly before elective EVAR, minimizing the risk of myocardial injury perioperatively.

Increased levels of hs-cTnI are found in myocardial injury, which could be caused by various reasons, such as myocarditis, HF, severe sepsis, operational stress [23,24,25,26]. However, diagnosis of myocardial infarction is set not only by increased levels of hs-cTnI, but also by rising values according to various available algorithms, clinical findings such as chest pain and ECG alterations. In our study only two patients (1.9%) presented MACEs, while 18 patients (17.3%) presented increased levels of hs-cTnI. The majority of these patients presented subclinical myocardial injury. Increased levels of hs-cTn are strong predictors of future short- and long-term cardiovascular events. Regarding the short-term events, high values of hs-cTnI have been related to increased risk for 30-day MACEs, 30-day mortality and readmission [27,28], while numerous strategies have been developed to rapidly rule out ACS using hs-cTn, thus contributing to shorter hospital stay [29]. Although in our study we did not record any deaths during the hospitalization of the patients, the hospital stay was prolonged for patients with MACEs, while the rest of the patients with increased hs-cTnI levels were advised to be reassessed and have a closer follow-up by their cardiologists. Increased levels of hs-cTnI are also long-term predictors of cardiovascular mortality, MACEs, HF and in general, structural and functional deterioration of the heart [24,30,31].

Regarding the limitations of the present study, we should mention the small number of patients enrolled in one center, although the inclusion was determined in a prospective and consecutive way. The level of evidence is not the highest possible according to the design of the study. As a result, the findings should be generalized with caution. However, the inclusion criteria contributed to a homogeneous patient group. The type of anesthesia administered to each patient was decided by the anaesthesiologist. This fact could lead to a selection bias due to lack of randomisation in our study regarding the findings between the two anesthesia patient groups. Additionally, myocardial injury after an operation could be attributed to various factors, which were not investigated in the present study. Fixed hs-cTn time points may miss peak values and data from postoperative echocardiography and other functional tests (when these were conducted by cardiologists during assessment of myocardial injury patients) were not recorded as per protocol. However, the statistical analysis provided relatively reliable results. On the other hand, myocardial injury could be present even after the second postoperative day after EVAR, which was not investigated in the present study. In addition, the mid- and long-term consequences of myocardial injury on the patients should be studied further. These outcomes are beyond the scope of the present study, but they warrant further investigation in future studies.

## 5. Conclusions

The present study is an observational prospective study investigating the causative effect of the duration of the operation, the type of anesthesia, the age, and the preoperative hemoglobin of patients on postoperative hs-cTnI levels after EVAR and consequently on post-EVAR myocardial injury. General anesthesia is associated with significantly higher hs-cTnI concentrations than regional anesthesia, with excess increases particularly evident at 24 h. Older age, longer surgery, and higher baseline hs-cTnI are associated with higher postoperative levels, while higher preoperative hemoglobin predicts lower levels. Understanding the factors that are related to myocardial injury during EVAR could contribute to the improvement in procedures in order to minimize the harmful effects of EVAR on heart function. Further larger studies with higher levels of evidence are needed to validate our findings and investigate the long-term effects of EVAR procedure on myocardial injury and heart function.

## Figures and Tables

**Table 2:** Heart comorbidities, rest comorbidities and medications.

**Heart comorbidities**	**Finding**
CAD, n (%)	36 (34.6)
Previous ACS, n (%)	20 (19.2)
Previous PCI, n (%)	23 (22.1)
Previous CABG, n (%)	7 (6.7)
HF, n (%)	2 (1.9)
AF, n (%)	10 (9.7)
**Other comorbidities**	**Finding**
Hypertension, n (%)	74 (71.2)
Dyslipidemias, n (%)	78 (75)
Diabetes mellitus, n (%)	19 (18.3)
COPD, n (%)	16 (15.4)
CKD, n (%)	70 (67.3)
Stroke, n (%)	15 (14.4)
**Medications**	**Finding**
Antiplatelet, n (%)	63 (60.6)
Anticogulant, n (%)	12 (11.5)
Statins, n (%)	68 (65.4)

n: number; CAD: coronary artery disease; ACS: acute coronary syndrome; PCI: percutaneous coronary intervention; CABG: coronary artery bypass graft surgery; HF: heart failure; AF: atrial fibrillation; COPD: chronic obstructive pulmonary disease; CKD: chronic kidney disease.

**Table 3:** Endograft properties and operational details.

**Endograft properties**	**Finding**
Manufacturer: Ankura, n (%)	71 (68.2)
Endurant II, n (%)	10 (9.7)
Gore Excluder, n (%)	9 (8.7)
Alto, n (%)	6 (5.8)
Jotec, n (%)	5 (4.8)
Zenith Alpha, n (%)	3 (2.9)
Extension to external iliac artery, n (%)	10 (9.7)
**Operation details**	**Finding**
Anesthesia: General, n (%)	72 (69.2)
Regional, n (%)	32 (30.8)
Preoperative hemoglobin, g/dL (±SD)	14.2 (±1.6)
Mean operation time, min (±SD)	92.6 (±25.4)
Mean radiation time, min (±SD)	7.4 (±4.7)
Mean contrast quantity, mL (±SD)	107.1 (±63.3)

n: number, g/dL: grams per deciliter; SD: standard deviation; min: minutes; mL: milliliters.

**Table 4:** Myocardial injury and MACE between the two patient groups.

**Myocardial Injury**	**Finding**
Subclinical (increased hs-cTnI without symptoms), n (%)	18 (17.3)
MACE, n (%)	2 (1.9)
Myocardial infraction, n (%)	2 (1.9)
Need for revascularization, n (%)	0
Death, n (%)	0

hs-cTnI: high-sensitivity cardiac troponin I, n: number, MACE: major adverse cardiovascular events.

**Table 5:** hs-cTnI levels at 6, 24 and 48 h postoperatively in the two anesthesia groups.

	Time	Anesthesia	n	Mean	SD	Min	Max	*p*	% Change to 6 h
Log hs-cTnI	At 6 h	General	72	0.9	0.4	0.6	2.0	<0.01	n/a
		Regional	32	0.7	0.3	0.6	1.8		n/a
	At 24 h	General	72	1.1	0.6	0.6	3.3	<0.01	89
		Regional	32	0.8	0.3	0.6	1.9		136
	At 48 h	General	47	1.2	0.5	0.6	3.0	<0.01	76
		Regional	28	0.9	0.4	0.6	2.7		92

Log hs-cTnI: log link of high-sensitivity troponin I; h: hours; n: number; SD: standard deviation, n/a: not applicable.

## Data Availability

The original contributions presented in this study are included in the article. Further inquiries can be directed to the corresponding author.

## Abbreviations

The following abbreviations are used in this manuscript:

EVAR	Endovascular aortic repair
hs-cTnI	High-sensitivity cardiac troponin I
AAA	Abdominal aortic aneurysm
OSR	Open aortic repair
ESVS	European Society of Vascular Surgery
CIA	Common iliac artery aneurysm
ESRN	End-stage renal disease
MACE	Major adverse cardiovascular events
BMI	Body mass index
CAD	Coronary artery disease
ACS	Acute coronary syndrome
PCI	Percutaneous coronary angioplasty
CABG	Coronary artery bypass graft surgery
HF	Heart failure
AF	Atrial fibrillation
COPD	Chronic obstructive pulmonary disease
CKD	Chronic kidney disease
